# Rotator cuff irreparability or failure of repair (re-tear): technical note on middle trapezius tendon transfer for reproduction of supraspinatus function

**DOI:** 10.1186/s40634-021-00426-y

**Published:** 2021-11-19

**Authors:** Amr Abdel-Mordy Kandeel

**Affiliations:** Department of Orthopedics & Traumatology, Faculty of Medicine, Menoufia University, Gamal Abdel-Nasser Street, Shebien El-kom, Menoufia Governorate Egypt

**Keywords:** Failed rotator cuff repair, Irreparable rotator cuff tear, Tendon transfer around the shoulder, Trapezius tendon transfer, Rotator cuff re-tear, Rotator cuff tear

## Abstract

**Purpose:**

Based on its close anatomic features and nearly-collinear force vector to those of supraspinatus muscle, the current article describes a technique of middle trapezius tendon transfer for reproduction of supraspinatus function in the context of rotator cuff irreparability/re-tear management.

**Methods:**

While seating the patient in beach-chair position, arthroscopic gleno-humeral examination and sub-acromial decompression are initially performed. Hamstring tendons are harvested and fashioned as flattened quadruple sheet. Through McKenzie approach, infraspinatus and subscapularis tendons are repaired. Then, medial half of middle trapezius insertion tendon is harvested from most medial 5-6 cm of the scapular spine. Through McKenzie approach, hamstring sheet is retrieved via a sub-trapezius/sub-acromial corridor from the scapular wound. Hamstring sheet is re-attached to cuff footprint by double row/suture bridge repair configuration. While retracting the scapula and placing gleno-humeral joint in 45^O^-abduction/45^O^-external rotation, hamstring sheet is re-attached to released middle trapezius tendon by non-absorbable sutures. Finally, tendon reconstruct is dynamically-tested in different positions of range of motion.

**Results:**

Transfer of medial portion of middle trapezius insertion tendon (lengthened by interposition hamstring tendon sheet) to cuff footprint was technically feasible. Dynamic testing showed smooth sub-acromial gliding motion of the tendon reconstruct.

**Conclusion:**

For reproduction of supraspinatus function, hamstring tendon augmented-middle trapezius tendon transfer to cuff footprint heralds a number of technical and biomechanical advantages; thus offering a potential effective modality of cuff irreparability/re-tear management in relatively young patients of high functional demands. However, current description should be investigated in further biomechanical and clinical studies to validate its long-term outcomes.

**Supplementary Information:**

The online version contains supplementary material available at 10.1186/s40634-021-00426-y.

## Introduction

In current orthopedic practice, postero-superior rotator cuff (RC) irreparability and re-tear remain challenging conditions for which tendon transfer of latissimus dorsi represented, over decades, a commonly-exercised management modality, notably, in young active patients of high functional demands [6].

.In 2009, Elhassan et al. described the technique of lower trapezius tendon transfer (LTTT) for management of traumatic brachial plexus palsy. Later in 2016, LTTT was investigated; however, in management of irreparable postero-superior RC deficiency yielding satisfactory outcomes in terms of shoulder functional scoring and restoration of external rotation (ER) provided that preoperative active forward flexion (FF) was more than 80^O^. The latter perquisite can be attributed to inability of this transfer to reproduce supraspinatus (SSP) function [3, 5].

.For reproduction of SSP function; middle trapezius tendon transfer (MTTT) might offer a number of potential biomechanical (e.g., almost collinear force vector with that of SSP), biological (e.g., abundant blood supply) and technical (e.g., simplified tendon harvest/convenient room of SSP fossa for gliding motion of tendon reconstruct) advantages which in turn are to result in almost re-normalized gleno-humeral (GH) kinematics and satisfactory clinical outcomes especially with regard to restoration of active GH elevation [8, 13, 14, 18].

.In a novel cadaveric technical description, Moroder et al. released the middle trapezius tendon from its acromial (lateral) attachment in order to be transferred to the footprint of a simulated SSP tear; concluding that insufficient length and dynamic mechanical block (against the acromion) of the transferred tendon remain questionable possibilities specifically in protracted-scapular position [13].

**Fig. 1:**
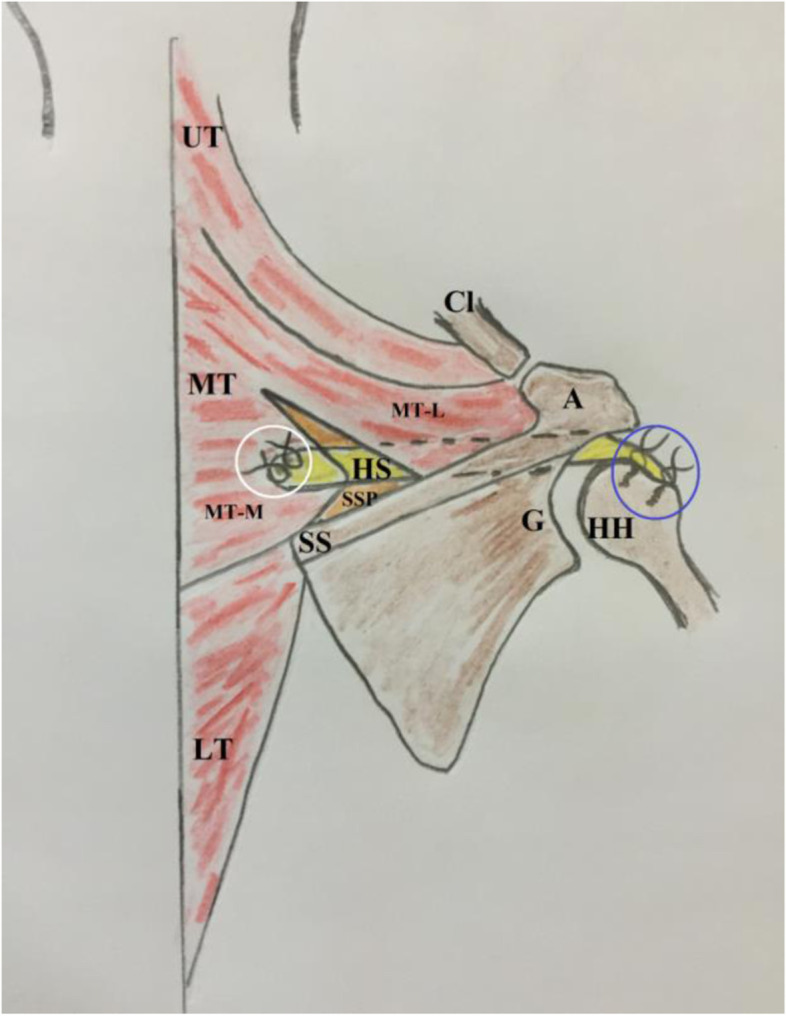
demonstrates technical principle of currently reported technique of middle trapezius tendon transfer for reproduction of supraspinatus function in the right shoulder. In this technique, the middle trapezius tendon is partially (medially) released (i.e. from medial half of the scapular spine), augmented/lengthened by an interposition fashioned sheet of hamstring tendon autograft using non-absorbable sutures (marked in white circle), and transferred in a sub-trapezius/sub-acromial corridor to the humeral head where it is re-attached in double-row suture configuration (marked in blue circle) to the footprint of supraspinatus tendon for management of rotator cuff irreparability/re-tear; A, acromion; C, coracoid; Cl, clavicle (lateral end); G, glenoid, HH, humeral head; HS, hamstring sheet; LT, lower trapezius; MT, middle trapezius; MT-L, middle trapezius (lateral insertion); MT-M, middle trapezius (medial insertion); SS, scapular spine; SSP, supraspinatus muscle (atrophied)

.Based on potential advantages of MTTT and to negate questionable remarks of transfer of acromial (lateral) portion of the middle trapezius insertion tendon, the current note reports a technique, whereby the middle trapezius tendon is partially (medially) released (i.e. from medial half of the scapular spine), augmented/lengthened by an interposition fashioned sheet of hamstring tendon autograft and transferred in a sub-trapezius/sub-acromial corridor to the humeral head where it is re-attached in double-row suture configuration to footprint of the RC (i.e. SSP) for management of RC irreparability/re-tear. Figure 1 demonstrates technical principle of the currently reported technique.

## Operative technique

Current technical note was approved by the Institutional Review Board in October, 2020 as a reconstructive procedure in relatively young active patients with irreparable RC tears or with failure of RC repair (i.e. re-tear).

### Setup

**Fig. 2:**
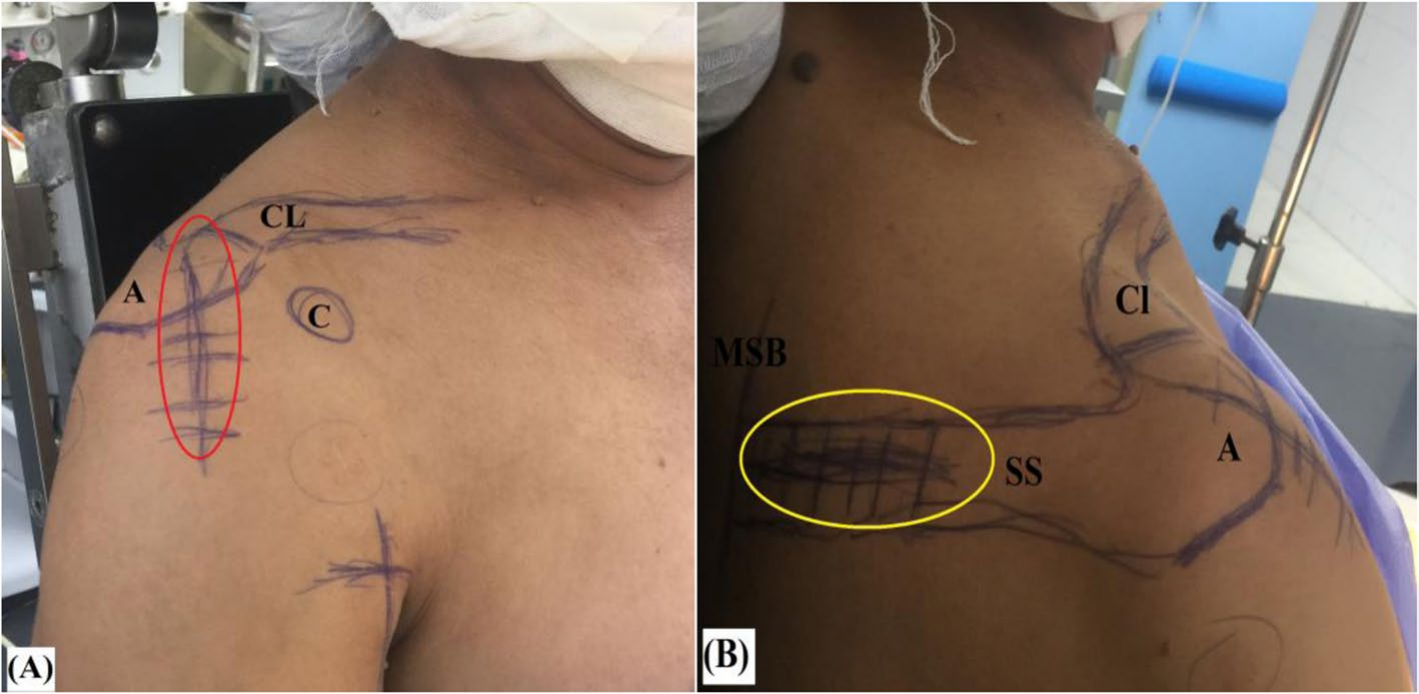
**A**, **B** demonstrate pen-marked soft-tissue and bony anatomic landmarks, McKenzie approach (marked in red oval), and scapular spine approach (marked in yellow oval) of currently reported technique of middle trapezius tendon transfer in the right shoulder; **A,** anterior aspect of the shoulder. **B,** posterior aspect of the shoulder; A, acromion; C, coracoid process; Cl, clavicle (lateral end); MSB, medial scapular border; SS, scapular spine

Following general anesthesia and prophylactic antibiotic administration (i.e., intravenous 1 g of Meropenem), the patient is seated in beach-chair position and related shoulder anatomic landmarks are pen-marked. Then, operated shoulder is examined under anesthesia for passive range of motion (ROM) to exclude diagnosis of concurrent frozen shoulder. Figure 2A, −B demonstrate pen-marked soft-tissue and bony anatomic landmarks and surgical approaches of the currently reported technique.

### Diagnostic arthroscopic gleno-humeral examination

Through viewing standard posterior GH and lateral sub-acromial portals, GH joint is examined to confirm diagnosis of RC irreparability/re-tear (based on criteria of massive retracted [i.e. to the glenoid] tear with poor tissue quality of the tendon stump), to ensure intact/reparable subscapularis (SSC), and to exclude concurrent intra-articular pathology (e.g., SLAP lesion, arthritic changes).

### Arthroscopic sub-acromial decompression

While viewing from the posterior GH portal, the scope is then swept over the torn cuff for improved visualization during sub-acromial decompression performed via lateral sub-acromial portal. This decompression includes 4 cardinal steps of bursectomy, coraco-acromial ligament release, anterior acromioplasty and debridement of arthritic acromio-clavicular joint.

### Hamstring tendon autograft harvesting

Under tourniquet, hamstring tendons are harvested through 5 cm long oblique skin incision placed midway between tibial tuberosity and midline of antero-medial aspect of proximal part of the ipsilateral tibia. Sub-cutaneous tissue is incised in line with skin incision and swept off by sponge gauze. Sartorius fascia is incised along its fibers to facilitate identification of pes anserinus. Tendons of gracilis and semitendinosus are sequentially grabbed by a curved artery clamp, freed from related expansions and harvested using an open/closed tendon stripper. Common insertion of both tendons is then detached with a part of related periosteum to get a tendon graft of about 22-24 cm in length.

### Hamstring tendon autograft fashioning

**Fig. 3:**
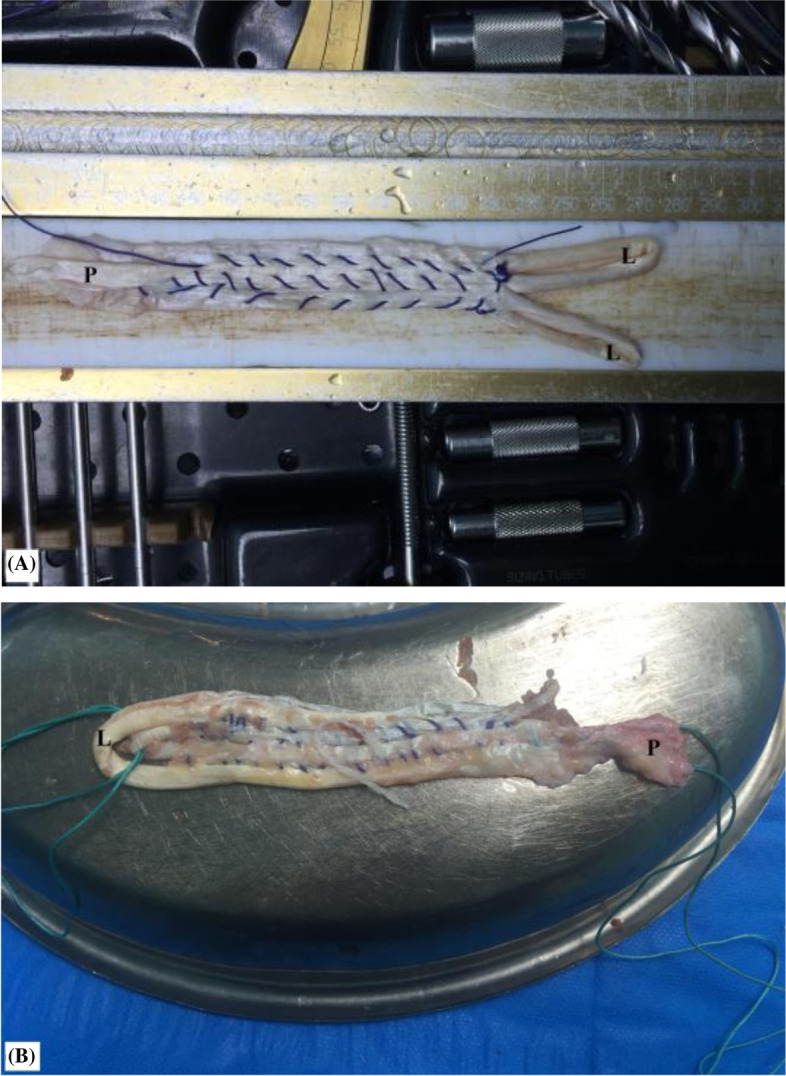
**A, B** demonstrate different patterns of sheet fashioning of harvested hamstring tendons to be used as an interposition graft in currently reported technique of middle trapezius tendon transfer; L, looed end; P, periosteal end

Afterwards, each harvested tendon is folded over itself to have a flattened quadruple sheet of a length not less than 12 cm. For re-enforced sheet-fashioning, folded tendon parts are side-by-side sutured to each other by #2 absorbable sutures (Vicryl, Ethicon, Cincinnati, OH, USA) over 8 cm middle segment of the sheet leaving the periosteal (2 cm) and looped (2 cm) tendon ends un-sutured. Figure 3-A, −B demonstrate different patterns of sheet fashioning of harvested hamstring tendons. Pearls and pitfalls of the currently reported technique are summarized in Table 1.

**Table 1:** Summarizes pearls and pitfalls of currently reported technique of middle trapezius tendon transfer; *EAU* examination under anesthesia, *GH* gleno-humeral, *LHB* long head of biceps, *ISP* infraspinatus, *RC* rotator cuff, *SSC* subscapularis, *SSP* supraspinatus

**Pearls:**	
*Setup -Beach-chair position with accessible back of the shoulder (notably, medially up to the dorsal spine) -EAU (for exclusion of frozen shoulder)	
*Diagnostic arthroscopic GH examination	
*Arthroscopic sub-acromial decompression	
*Hamstring tendon autograft harvesting -Common insertion of harvested gracilis and semitendinosus tendons is detached with a part of related periosteum to get a tendon graft of about 22-24 cm in length.	
*Hamstring tendon autograft fashioning -Each harvested tendon is folded over itself to have a quadruple sheet of a length not less than 12 cm. -For re-enforcement/sheet-fashioning, folded tendons are side-by-side sutured by # 2 absorbable sutures	
*McKenzie approach -Remnant sub-acromial bursa is excised by to improve visualization during next steps -Footprint of RC is well-prepared to improve biology for healing of future tendon reconstruct. -Tension-free anatomic/partial/medialized RC (ISP+/−SSP) repair is performed -Free suture limbs of RC repair are left uncut for later suturing of the hamstring sheet to the repaired RC -When concurrently torn, SSC is anatomically repaired to its footprint -Soft-tissue tenodesis of tenotomized LHB to repaired RC	
*Middle trapezius tendon release -Skin overlying medial half (5-6 cm) of the scapular spine is transversely incised -Medial portion (5-6 cm) of middle trapezius insertion is identified, stay-sutured, and released (with related periosteum) from scapular spine by diathermy in a lateral-to-medial direction till medial scapular border -To improve its excursion, undersurface of released tendon is bluntly dissected (by sweeping finger) from underlying atrophic SSP	
*Sub-trapezius/sub-acromial passage of the hamstring sheet -A long straight artery clamp is passed via McKenzie approach deep to the acromion to the scapular wound to establish a sub-trapezius/sub-acromial corridor to retrieve hamstring sheet from the scapular to the humeral sides.	
*Re-attachment of the hamstring sheet on the humeral side -Periosteal end of the hamstring sheet is trans-osseously sutured to anatomic footprint of RC (notably of SSP) by #5 non-absorbable sutures and to the repaired RC by uncut free suture limbs of suture anchors used for RC repair.	
*Re-attachment of the hamstring sheet on the scapular side -While retracting the scapula and placing GH joint in 45^O^-abduction/45^O^-external rotation position, hamstring sheet is sutured (in Pulvertaft/ side-to-side fashion) to released middle trapezius tendon by #5 non-absorbable sutures	
*Dynamic testing of the construct -For: integrity and smooth sub-acromial gliding motion of the tendon reconstruct -By: placing GH joint in different positions of range of motion and by axial loading of tendon reconstruct	
**Pitfalls:**	
-Suture-tagging of medial insertion of middle trapezius (prior to harvesting) is to facilitate its release and excursion testing -Medial insertion of middle trapezius should be released in a sub-periosteal fashion to harvest tendon stump able to withstand Pulvertaft/side-to-side suturing; otherwise; integrity of the tendon reconstruct is to be compromised -Release of medial portion of middle trapezius should not exceed medial scapular border to avoid spinal accessory nerve injury -Short harvested medial insertion of middle trapezius is to subject the tendon reconstruct to excessive tension -Capacious sub-trapezius/sub-acromial corridor is to ensure free smooth sub-acromial gliding motion of the tendon reconstruct -Adequate length (for future attachment on both humeral and scapular sided) of the hamstring sheet should be evaluated following its sub-trapezius/sub-acromial passage	

### McKenzie approach

Through 5 cm long skin incision centered over antero-lateral corner of the acromion, subcutaneous tissue is incised and swept off, and deltoid raphe between its anterior and middle thirds is longitudinally incised and retracted by self-retaining retractor. Remnant sub-acromial bursa is excised by a diathermy probe to improve visualization during next steps.

**Fig. 4:**
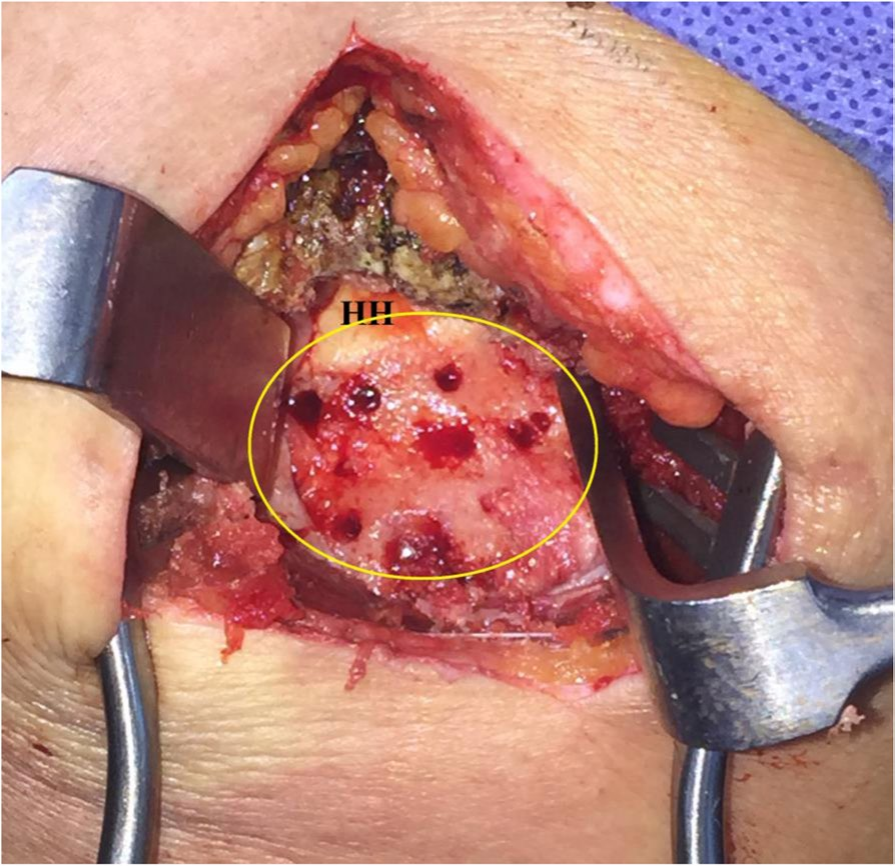
demonstrates prepared footprint (marked in yellow oval) of the rotator cuff via McKenzie approach for future anatomic/partial/medialized repair of the rotator cuff and re-attachment of fashioned hamstring sheet in currently reported technique of middle trapezius transfer in the right shoulder; HH, humeral head

Then, footprint of torn RC is defined, debrided, minimally decorticated by a curette and repeatedly-holed by an awl in order to improve local biology for healing of future tendon reconstruct. Figure 4 demonstrates prepared footprint of the rotator cuff via McKenzie approach.

**Fig. 5:**
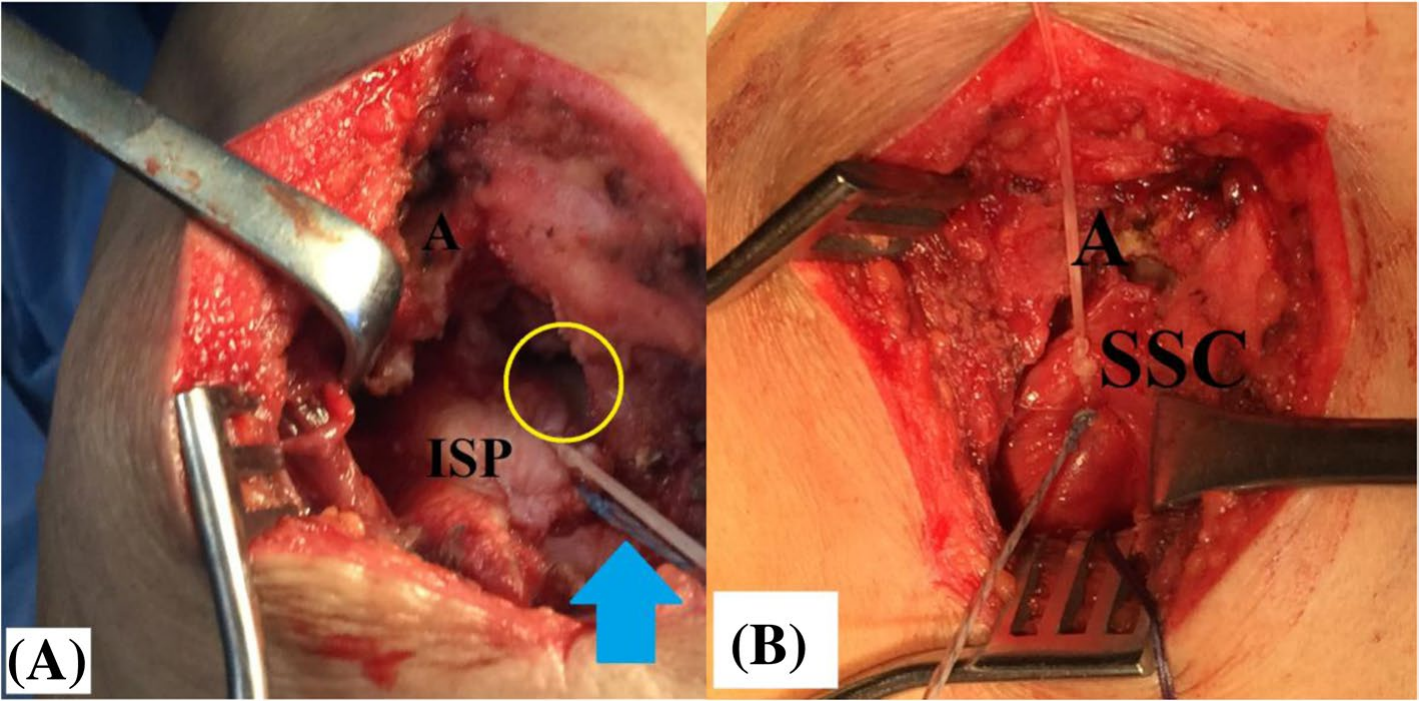
**A**, **B** demonstrate partially-repaired postero-superior rotator cuff and anatomic repair of subscapularis in the setting of currently reported technique of middle trapezius tendon transfer in the right shoulder. **A,** repaired infraspinatus tendon leaving the retracted supraspinatus tendon un-repaired, the yellow oval-marked humeral head partially-uncovered, and the blue arrow-marked repair sutures uncut for future re-attachment of the hamstring sheet to the repaired rotator cuff. **B,** When concurrently torn, subscapularis is anatomically repaired to its footprint to re-establish gleno-humeral force couple mechanism in the transverse plane; A, acromion; ISP, infraspinatus; SSC, subscapularis

Afterwards, long head of biceps (LHB) tendon is released from its bicipital groove, stay-sutured and tenotomized from its labral origin for future soft tissue tenodesis to the repaired cuff. Torn RC is then released, reduced to its footprint, and (anatomically/partially/medially) repaired by 2 double-loaded suture anchors (2.9 mm JuggerKnot® All-Suture Anchors, Zimmer Biomet, Warsaw, IN, USA). Following repair, free suture limbs are left uncut for later suturing of the hamstring sheet to the repaired RC. When concurrently torn, SSC is anatomically repaired to its footprint using an additional suture anchor. Figure 5-A, −B demonstrate partially-repaired postero-superior rotator cuff and anatomic repair of subscapularis in the setting of currently reported technique.

### Middle trapezius tendon release

**Fig. 6:**
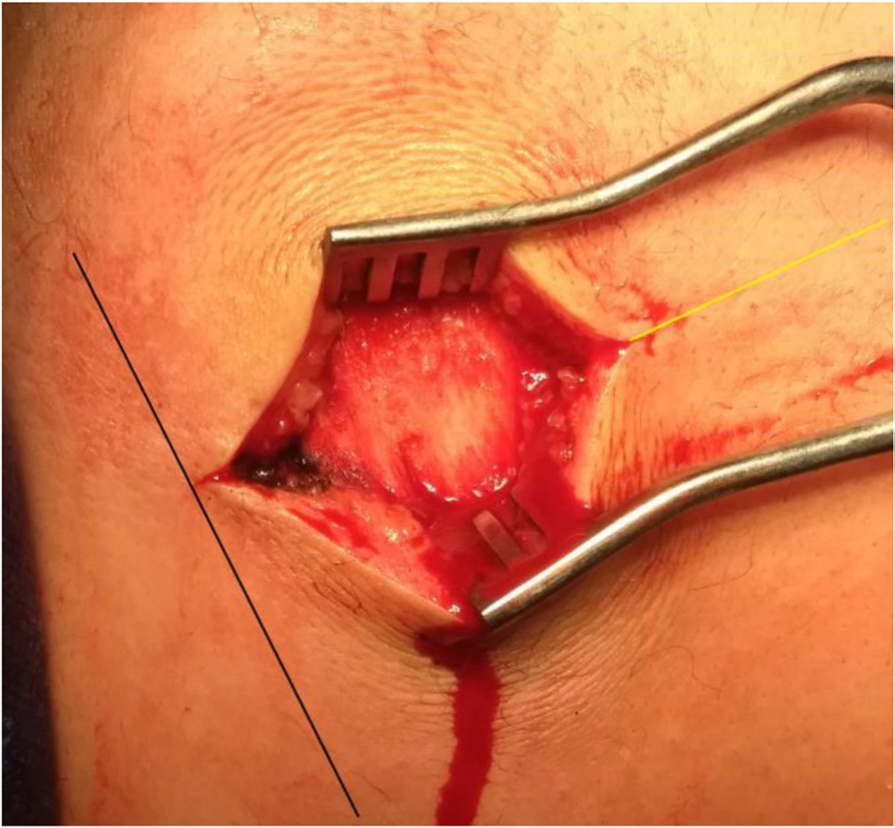
demonstrates skin incision for approaching medial portion of the middle trapezius insertion tendon in the right shoulder. Starting from the black line-marked medial scapular border and extending laterally for a distance of 5-6 cm, the skin overlying medial half of the yellow line-marked scapular spine is horizontally incised

Starting from the medial scapular border and extending laterally for a distance of 5-6 cm, the skin overlying medial half of the scapular spine is horizontally incised. Then, subcutaneous tissue is swept by sponge gauze till exposing medial half of upper lip of the scapular spine into which medial part of the middle trapezius tendon is inserted. Figure 6 demonstrates skin incision for approaching medial portion of the middle trapezius insertion tendon.

Thereafter, medial portion of the middle trapezius insertion tendon is identified and tagged with stay-suture of #2 absorbable sutures (Vicryl, Ethicon, Cincinnati, OH, USA) which can also be used as traction suture to facilitate tendon release (with a part of related periosteum) from most medial 5-6 cm of the scapular spine by a diathermy probe starting laterally and proceeding medially till the medial scapular border.

**Fig. 7:**
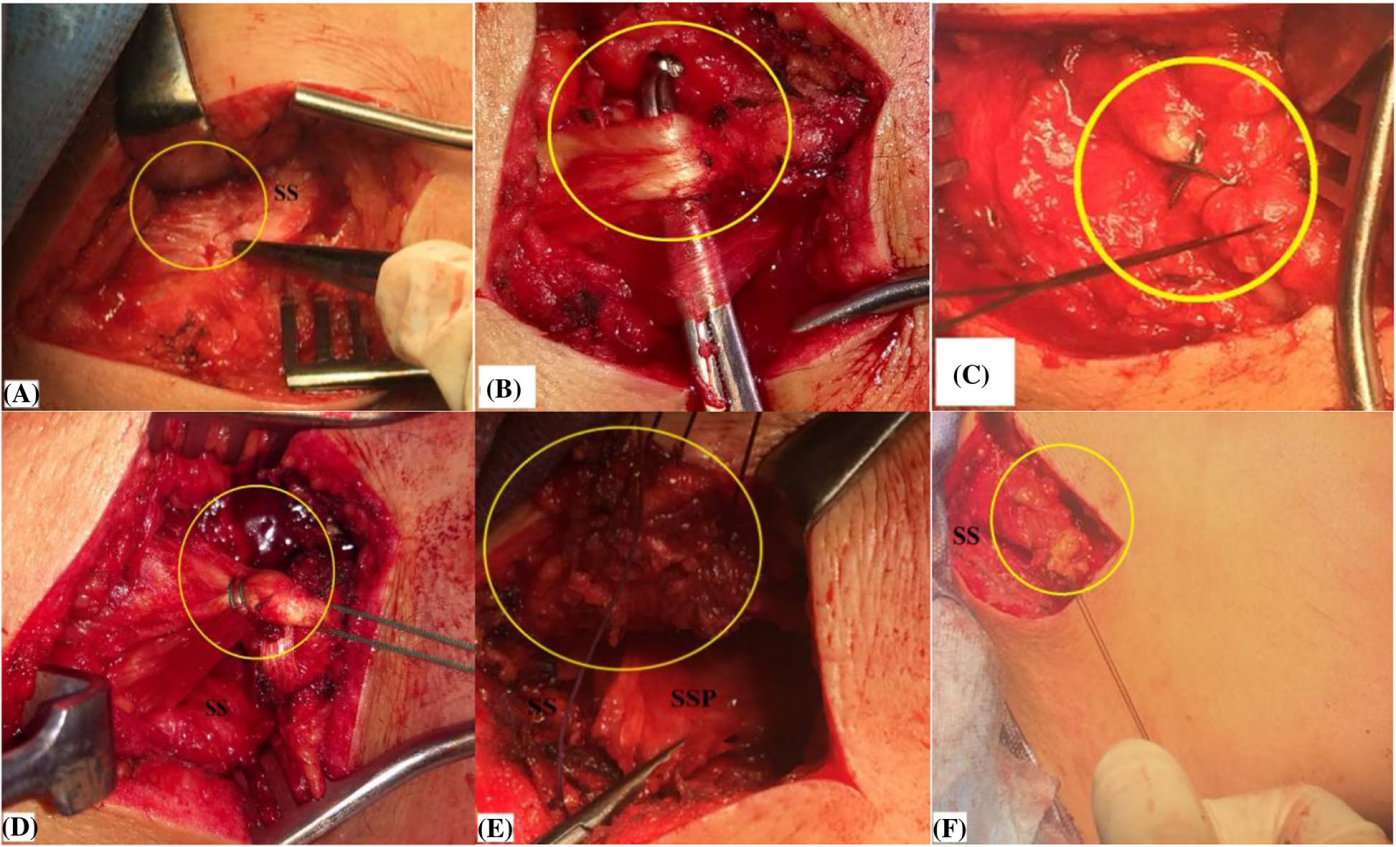
**A, −B, −C, −D, −E, −F** demonstrate release of medial portion (marked in yellow circle) of the middle trapezius insertion tendon in the right shoulder. **A and B,** identification of the tendon insertion into the scapular spine; **C,** suture tagging of the tendon to facilitate next steps of tendon release and excursion testing; **D,** tendon release in a lateral-to-medial direction up to the medial scapular border; **E,** blunt dissection of the released tendon from underlying atrophied supraspinatus muscle; **F,** testing the released tendon for free and adequate excursion; SS, scapular spine; SSP, supraspinatus muscle

In order to improve tendon excursion, undersurface of the middle trapezius muscle is released from underlying atrophied SSP muscle by blunt (i.e. finger sweeping) dissection up to the medial scapular border. Figure 7-A, −B, −C, −D, −E, −F demonstrate release of medial portion of the middle trapezius insertion tendon.

### Sub-trapezius/sub-acromial passage of the hamstring sheet

**Fig. 8:**
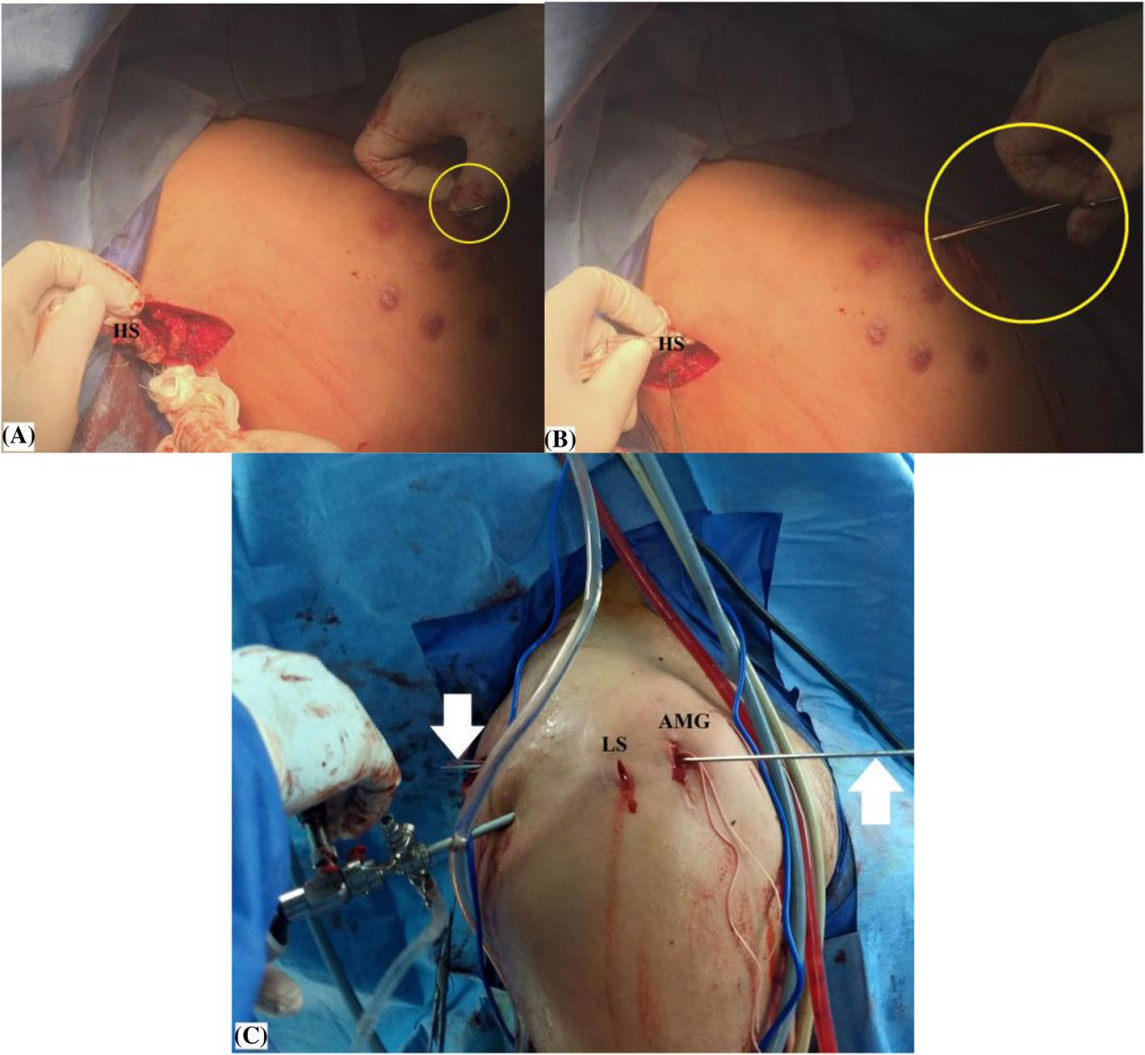
**A, −B, −C** demonstrate sub/trapezius sub-acromial passage of the hamstring sheet in currently reported technique of middle trapezius tendon transfer in the right shoulder. **A,** a long straight artery clamp (marked in yellow circle) passed through McKenzie approach under the acromion to the scapular wound to establish a sub-trapezius/sub-acromial corridor; **B,** sub-trapezius/sub-acromial shuttling of the hamstring sheet aided by the passed long straight artery clamp (marked in yellow circle); or alternatively by; **C,** an anterior cruciate ligament guide pen (marked by white arrows); the latter is valid for both open and arthroscopic-assisted modalities of currently reported technique; AMG, anterior mid-glenoid portal; HS, Hamstring sheet; LS, lateral sub-acromial portal

Using a long straight artery clamp passed through McKenzie approach under the acromion to appear at the scapular wound; a sub-trapezius/sub-acromial corridor is established in order to retrieve the hamstring sheet from the scapular to the humeral sides. Figure 8-A, −B, −C demonstrate sub-trapezius/sub-acromial passage of the hamstring sheet in the currently reported technique.

**Fig. 9:**
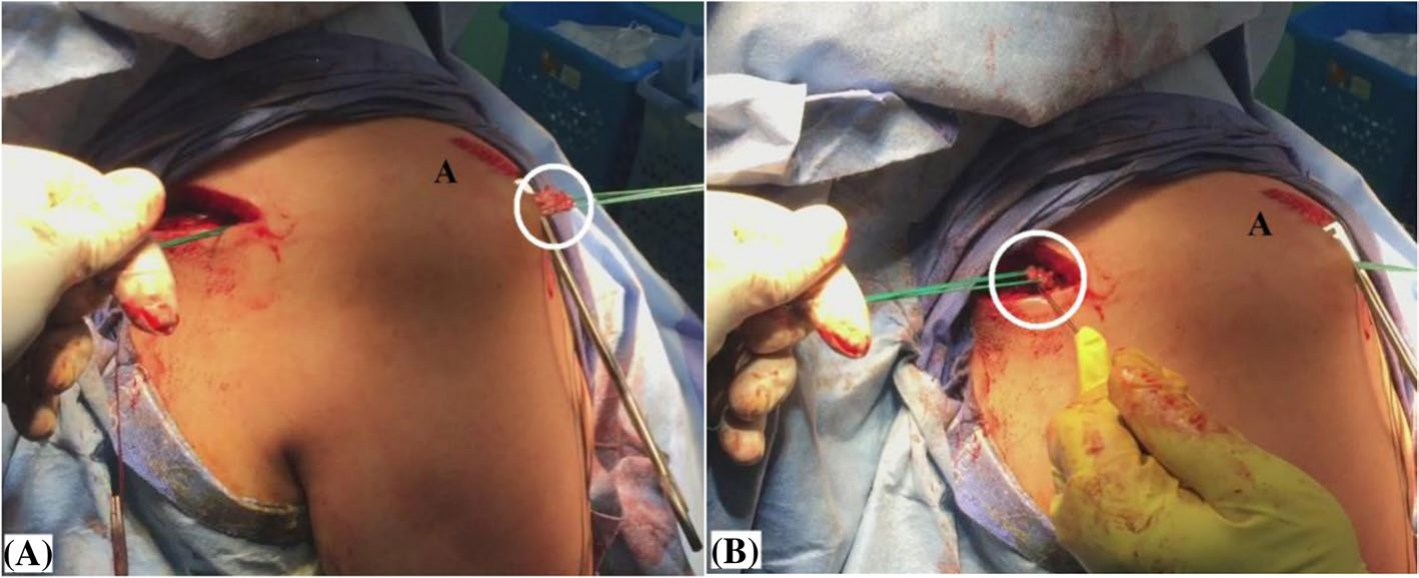
**A, −B** demonstrate checking of adequate length of the hamstring sheet (marked in white circle) for future re-attachment in currently reported technique of middle trapezius tendon transfer in the right shoulder; **A,** on the humeral side; **B,** on the scapular side; A, acromion.

By this stage, it is essential to ascertain free smooth sub-acromial gliding motion and adequate length (for future re-attachment on both humeral and scapular sides) of the hamstring sheet. Figure 9-A, −B demonstrate checking of adequate length of the hamstring sheet in the currently reported technique.

### Re-attachment of the hamstring sheet on the humeral side

**Fig. 10:**
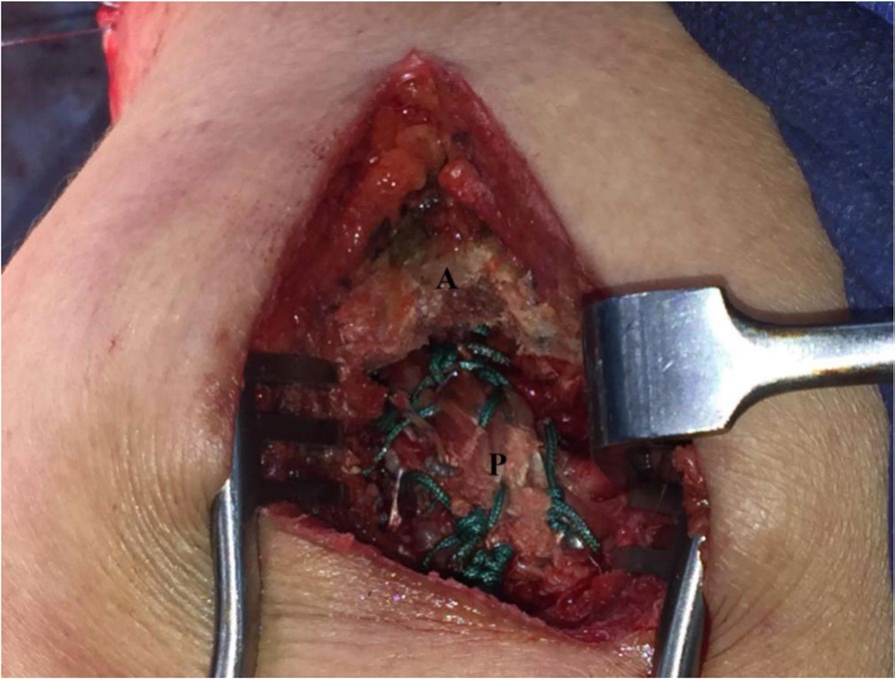
demonstrates sutured periosteal end of the hamstring sheet to anatomic footprint of the rotator cuff, and additionally to the repaired rotator cuff via McKenzie approach in currently reported technique of middle trapezius tendon transfer in the right shoulder; A, acromion; P, periosteal end of the hamstring sheet

Thereafter, periosteal end of the hamstring sheet is trans-osseously sutured to the anatomic RC footprint notably of SSP and infraspinatus (ISP) tendons using #5 non-absorbable sutures (Ethibond*Excel, Ethicon, Cincinnati, OH, USA). In addition, this periosteal end is sutured to the repaired RC taking advantage of uncut free suture limbs of the suture anchors used for RC repair. Figure 10 demonstrates sutured periosteal end of the hamstring sheet to anatomic footprint of the rotator cuff, and additionally to the repaired rotator cuff.

### Re-attachment of the hamstring sheet on the scapular side

**Fig. 11:**
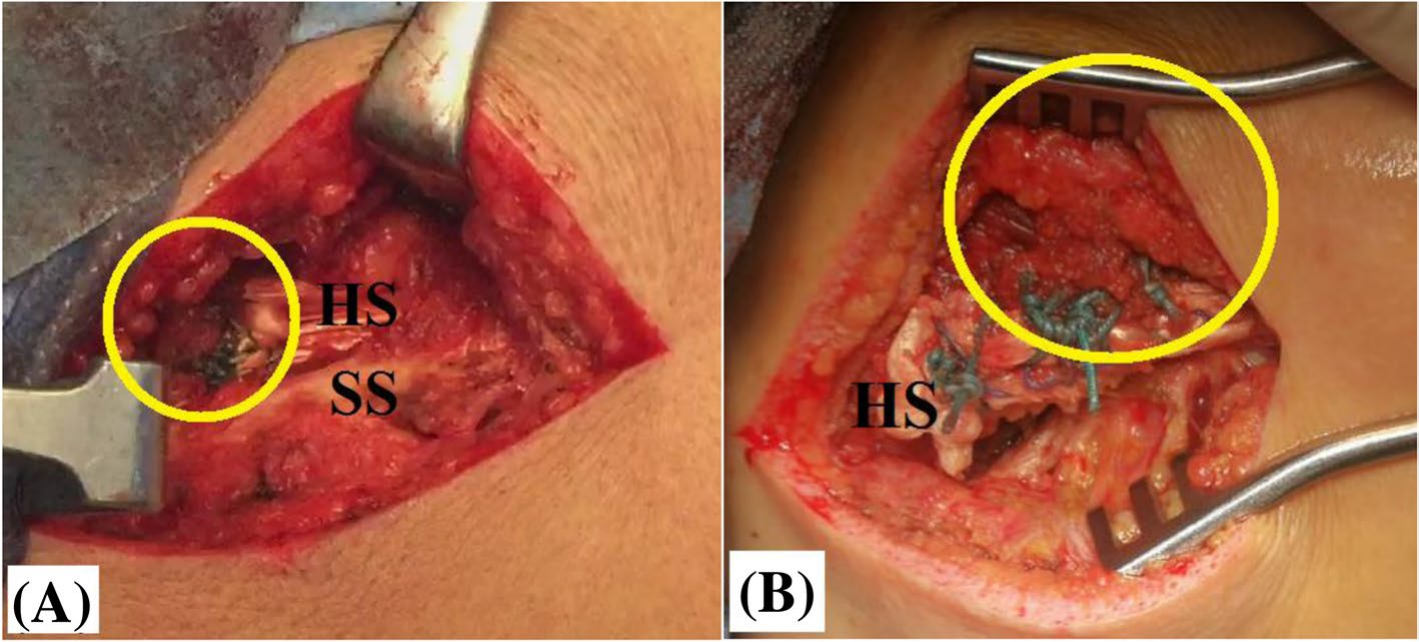
**A, −B** demonstrate sutured looped ends of the hamstring sheet to the released middle trapezius tendon (marked in yellow circle) by #5 non-absorbable sutures in currently reported technique of middle trapezius tendon transfer in the right shoulder; **A,** Pulvertaft fashion of suturing; **B**, side-to-side fashion of suturing; HS, hamstring sheet; SS, scapular spine

While retracting the scapula and placing GH joint in 45^O^-abduction/45^O^-external rotation position, looped ends of the hamstring sheet is sutured (in Pulvertaft/side-to-side fashion) to the released middle trapezius tendon by #5 non-absorbable sutures (Ethibond*Excel, Ethicon, Cincinnati, OH, USA). Figure 11A, −B demonstrate sutured looped ends of the hamstring sheet to the released middle trapezius tendon.

### Dynamic testing of the construct

**Fig. 12:**
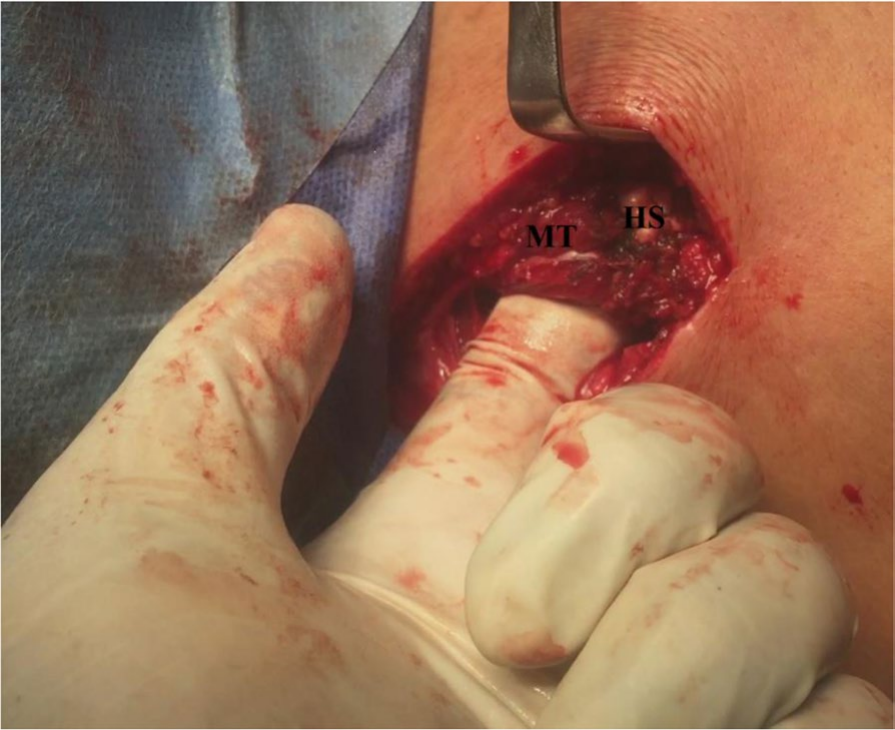
demonstrate tested integrity of the tendon reconstruct by axial loading of the transferred middle trapezius tendon in the right shoulder; HS, hamstring sheet; MT, transferred middle trapezius tendon

Finally, integrity and smooth sub-acromial gliding motion of the tendon reconstruct are dynamically tested by placing GH joint in different ROM positions and by axial loading (i.e. via longitudinal traction) of transferred middle trapezius tendon. Figure 12 demonstrates tested integrity of the tendon reconstruct by axial loading of transferred middle trapezius tendon.

For more illustration, technical steps of currently reported technique of middle trapezius tendon transfer are demonstrated in Supplemental Video 1.


Additional file 1: **Video 1.** The current note describes a technique of middle trapezius tendon transfer for management of rotator cuff irreparability/re-tear in relatively young active patients of high functional demands. In currently-reported technique, medial portion of the middle trapezius insertion tendon is partially released from the scapular spine, augmented/lengthened by an interposition fashioned sheet of hamstring tendon autograft and transferred in a sub-trapezius/sub-acromial corridor to the humeral head where it is re-attached in double-row suture configuration to footprint of the rotator cuff. Technical description of the current note includes the following steps: 1-Following general anesthesia and prophylactic antibiotic administration, the patient is seated in beach-chair position, related shoulder anatomic landmarks are pen-marked, and operated shoulder is examined under anesthesia for passive range of motion to exclude diagnosis of concurrent frozen shoulder. 2-Diagnostic arthroscopic gleno-humeral examination is performed to confirm diagnosis of rotator cuff irreparability/re-tear, to ensure intact/reparable subscapularis, and to exclude concurrent intra-articular pathology (e.g. SLAP lesion, arthritic changes). 3-Arthroscopic sub-acromial decompression is then carried out. 4-Thereafter, hamstring tendon autograft of gracilis and semitendinosus are harvested by tendon stripper and their common insertion is detached with a part of related periosteum to get a tendon graft of about 22-24 cm in length. 5-Then, harvested Hamstring tendons are fashioned by folding each harvested tendon over itself to have a flattened quadruple sheet of a length not less than 12 cm. For re-enforced sheet-fashioning, folded tendon parts are side-by-side sutured to each other by #2 absorbable sutures. 6-Through McKenzie approach, remnant sub-acromial bursa is excised by a diathermy probe to improve visualization during next steps, footprint of torn rotator cuff is debrided and minimally decorticated in order to improve local biology for healing of future tendon reconstruct, long head of biceps tendon is stay-sutured and tenotomized from its labral origin for future soft tissue tenodesis to the repaired cuff and torn rotator cuff is (anatomically/partially/medially) repaired by 2 double-loaded suture anchors. Following cuff repair, free suture limbs are left uncut for later suturing of the hamstring sheet to the repaired cuff. When concurrently torn, subscapularis is anatomically repaired to its footprint using an additional suture anchor. 7-Through a 5-6 cm transverse skin incision over medial half of the scapular spine, medial portion of the middle trapezius insertion tendon is identified and tagged with stay-suture of #2 absorbable sutures which can also be used as traction suture to facilitate tendon release (with a part of related periosteum) from most medial 5-6 cm of the scapular spine by a diathermy probe starting laterally and proceeding medially till the medial scapular border. In order to improve tendon excursion, undersurface of the middle trapezius muscle is released from underlying atrophied supraspinatus muscle by blunt (i.e. finger sweeping) dissection up to the medial scapular border. 8-Thereafter, the hamstring sheet is shuttled through a sub-trapezius/sub-acromial corridor: A long straight artery clamp is passed through McKenzie approach under the acromion to appear at the scapular wound to establish a sub-trapezius/sub-acromial corridor through which the hamstring sheet is retrieved from the scapular to the humeral sides by the same artery clamp/or by an anterior cruciate ligament guide pin. By this stage, it is essential to ascertain free smooth sub-acromial gliding motion and adequate length (for future re-attachment on both humeral and scapular sides) of the hamstring sheet. 9-On the humeral side, periosteal end of the hamstring sheet is trans-osseously sutured to the anatomic rotator cuff footprint notably of supraspinatus and infraspinatus tendons using #5 non-absorbable sutures. In addition, this periosteal end is sutured to the repaired cuff taking advantage of uncut free suture limbs of the suture anchors used for cuff repair. 10-On the scapular side, looped ends of the hamstring sheet is sutured (in Pulvertaft/side-to-side fashion) to the released middle trapezius tendon by #5 non-absorbable sutures (while retracting the scapula and placing gleno-humeral joint in 45^O^-abduction/45^O^-external rotation position). 11-Finally, integrity and smooth sub-acromial gliding motion of the tendon reconstruct are dynamically tested by placing gleno-humeral joint in different positions of range of motion and by axial loading (i.e. via longitudinal traction) of transferred middle trapezius tendon.

#### Postoperative rehabilitation

For 6 weeks, operated shoulder is placed in a standard shoulder sling preferably coupled with an abduction pillow. During these early postoperative weeks, isometric deltoid and active ROM exercises of the elbow are encouraged. Next, the sling is discarded to commence 2 weeks of pendulum and active-assisted ROM exercises of the operated shoulder. By 8 weeks postoperatively, patient starts a 16-week rehabilitation protocol of stretching, strengthening and neuromuscular re-education (proprioception, balance and coordination) exercises under supervision of the surgeon and physiotherapist. Return to work and overhead-related activities are allowed by 24 weeks postoperatively.

## Discussion

Within few years, trapezius tendon transfer has evolved as a paradigm shift in management of RC irreparability/re-tear. Initially, in 2009; Elhassan et al. introduced LTTT for restoration of shoulder ER in a patient with post-traumatic brachial plexus palsy [3].

.Meanwhile in 2011, Goutallier et al. disapproved upper trapezius tendon transfer (UTTT) for management of antero-superior RC deficiency in a prospective case series of 20 irreparable SSC tears [7].

.On other hand in 2016, Elhassan et al. reported satisfactory functional outcomes of LTTT for management of irreparable postero-superior RC tears [5].

.Since then, different open and arthroscopic technical modifications (in terms of graft options and methods of fixation) of LTTT have been described concluding significant postoperative improvement in ROM and strength of shoulder ER. The latter conclusion was partly explained by the collinear force vector of LTTT with that of ISP. Nevertheless, preoperative shoulder FF of <80^O^ remained a contraindication (i.e. poor prognostic factor) for LTTT due to its inability to reconstitute SSP function [4, 12, 16, 17].

.To overcome the latter limitation of LTTT, MTTT to SSP footprint is to exert a force vector almost collinear to that of SSP; so offering a biomechanical rationale for MTTT as a management option of irreparable SSP tear [8, 13].

.In 2015, a cadaveric study by Omid et al. redefined surgical anatomy of the 3 distinct (upper/middle/lower) segments of trapezius muscle showing that the middle trapezius has a broad insertion to the acromion and whole upper lip of the scapular spine [14].

.Later in a recent cadaveric study published in 2021, Moroder et al. demonstrated surgical feasibility of transfer of a part (i.e. acromial/lateral portion) of this broad insertion of the middle trapezius to the footprint of an excised SSP tendon pointing out that the pre-transfer average angles between middle trapezius tendon and SSP (i.e. 33° and 34° in the transverse and coronal planes respectively) changed into more collinear orientation (i.e. 30° and 24° in the transverse and coronal planes respectively) following transfer. However in this study, mechanical block of the transferred tendon against the acromion (in protracted-scapula position) was a concern [13].

.Based on potential biomechanical advantage of MTTT in management of irreparable postero-superior RC and to overcome mechanical block related to transfer of acromial (lateral) portion of the middle trapezius insertion tendon, the current note reports a technique in which medial 5 cm of scapular-spine insertion of the middle trapezius tendon is sub-trapezius/sub-acromially transferred to footprint of SSP and ISP.

As regards its indications, currently reported technique is described for management of postero-superior RC irreparability/re-tear in young active population of high functional demands. In addition, this technique might also be applied for true paralysis of SSP/ISP caused by isolated suprascapular nerve injury.

A point worth mentioning is that standard perquisites of tendon transfer (e.g. mobile non-arthritic GH joint) are still pre-conditioned for the current technical note. Indications and contraindications of the currently reported technique are summarized in Table 2.

**Table 2:** Summarizes indications and contraindications of currently reported technique of middle trapezius tendon transfer; *GH* gleno-humeral, *ISP* infraspinatus, *RC* rotator cuff, *SSC* subscapularis, *SSP* supraspinatus

**Indications:**	
*Irreparable RC tear with the following criteria: -Clinical findings of persistent shoulder pain and/or pseudo-paralysis -X-ray finding of decreased acromio-humeral distance (< 7 mm) -MRI findings of > grade-II Patte retraction, > grade-II Goutallier fatty infiltration and > grade-II Thomazeau SSP muscle atrophy -Intra-operative finding of poor soft tissue quality of torn tendon stump *RC re-tear with the following 6–9 months postoperative criteria: -Clinical findings of persistent shoulder pain and/or pseudo-paralysis -Ultra-sound findings of RC non-vascularization, discontinuity and/or retraction on dynamic testing -MRI findings of RC discontinuity and/or retraction *Isolated supra-scapular nerve injury	
**Contraindications:**	
*Absolute contraindications: -Advanced arthritis of GH joint -Trapezius muscle paralysis -Irreparable SSC -Irreparable ISP -Non-functioning deltoid muscle (e.g. dehiscence, axillary nerve injury) -Active infection *Relative contraindications: -History of infection following RC repair -Un-motivated patient for 6–9 months postoperative rehabilitation -Elderly patients (i.e. > 65 years old) -Shoulder stiffness	

Technically, the current description is much more simplified and less invasive when compared with its counterpart of Moroder et al.; as it requires release of a small part (i.e. 5 cm) rather than main bulk of the middle trapezius insertion. Technical differences of the currently reported technique from that of Moroder et al. are summarized in Table 3 [13].

**Table 3:** Summarizes technical differences of currently reported technique of middle trapezius tendon transfer from that of Moroder et al.; *AC* acromio-clavicular, *GH* gleno-humeral, *ISP* infraspinatus, *RC* rotator cuff, *SSP* supraspinatus

Technical Difference	Current technique	Technique of Moroder et al
*Decubitus*	Beach-chair	prone
*Anatomic landmarks*	Medial half of scapular spine	Clavicle, AC joint, acromion, and scapular spine
*Approach for trapezius harvesting*	5 cm skin incision over and parallel to medial half of the scapular spine	5 cm skin incision parallel to anterior border of the spino-acromial junction.
*Released insertion of middle trapezius*	Medial half of its scapular-spine insertion (via sub-periosteal dissection)	Acromial (lateral) insertion (via sub-periosteal dissection)
*Extensile harvesting of middle trapezius*	Feasible (in lateral direction) *Medially: limited by spinal accessory nerve	Feasible (in medial direction) *Laterally: limited by AC joint
*Split of fleshy middle trapezius*	Oblique split (along its fibers), (for 5 cm)	Longitudinal split, (for 3 cm)
*Release of fleshy middle trapezius from underlying SSP*	Blunt dissection (finger sweeping)	–
*Preparation of released tendon*	Sutured (in Pulvertaft/side-to-side fashion) to hamstring sheet	Sutured (in Krakow fashion)
*Interposed tendon graft*	Fashioned hamstring sheet	Not used (directly re-attaching transferred tendon to footprint of excised SSP)
*Corridor of transferred tendon*	Sub-trapezius/sub-acromial	Sub-acromial
*Fixation method to RC footprint*	Hamstring sheet is sutured to RC (SSP+/−ISP) footprint via trans-osseous sutures; and also, to repaired RC via direct suturing	Free limbs of Krakow sutures are passed within trans-osseous tunnels in RC (SSP) footprint
*Reproduction of SSP anatomic attachment* *To its footprint*	Feasible (via double-row/suture-bridge re-attachment of flattened periosteal end of hamstring sheet to SSP footprint)	–
*Scapular/GH position during reconstruction*	Retracted scapula & 45^O^-abduction/ 45^O^-external rotation of GH joint	Retracted scapula
*Room for gliding motion of the tendon reconstruct*	SSP fossa & SSP outlet	SSP outlet
*Mechanical block of the tendon reconstruct*	–	Reported in protracted-scapular position
*AC joint injury*	–	Potential risk
*Force vector of the transferred tendon*	Horizontally-oriented (medially-directed)	Vertically-oriented (superiorly-directed)

.In spite of this limited release, effective excursion of the released tendon portion can be achieved by wide blunt dissection of its undersurface from underlying SSP; however, this release should not be advanced beyond the medial scapular border to avoid injury of the spinal accessory nerve as the latter courses 1–2 cm medial to that border [8, 13].

.In addition, current technical description is in accordance with different reports advocating tendon graft augmentation of transferred trapezius segment to lengthen its released tendon up to the recipient site. Moroder et al. showed that in retracted-scapular position; acromial portion of the middle trapezius can be directly transferred to RC footprint without interposition tendon graft; however, this was not feasible in protracted-scapular position; concluding that need for interposition graft remains questionable and should be intraoperatively-considered on dynamic basis to negate excessive tension over the tendon reconstruct. Similarly, in a Newcastle shoulder model of virtual LTTT; Reddy et al. demonstrated necessity of interposition allograft to minimize risk of trapezius over-tensioning [8, 13, 15].

.Furthermore, effective reconstitution of SSP function by MTTT entails technical reproduction of anatomic RC footprint (notably SSP) during MTTT re-attachment. In the current technique, flattened periosteal end of the interposed hamstring sheet is to offer an insertion stump broad enough for global re-attachment to whole anatomic footprint of SSP and ISP. Besides, tendon-bone contact surface area and pressure of this re-attachment can be further maximized by double-row and suture-bridge repair techniques.

**Fig. 13:**
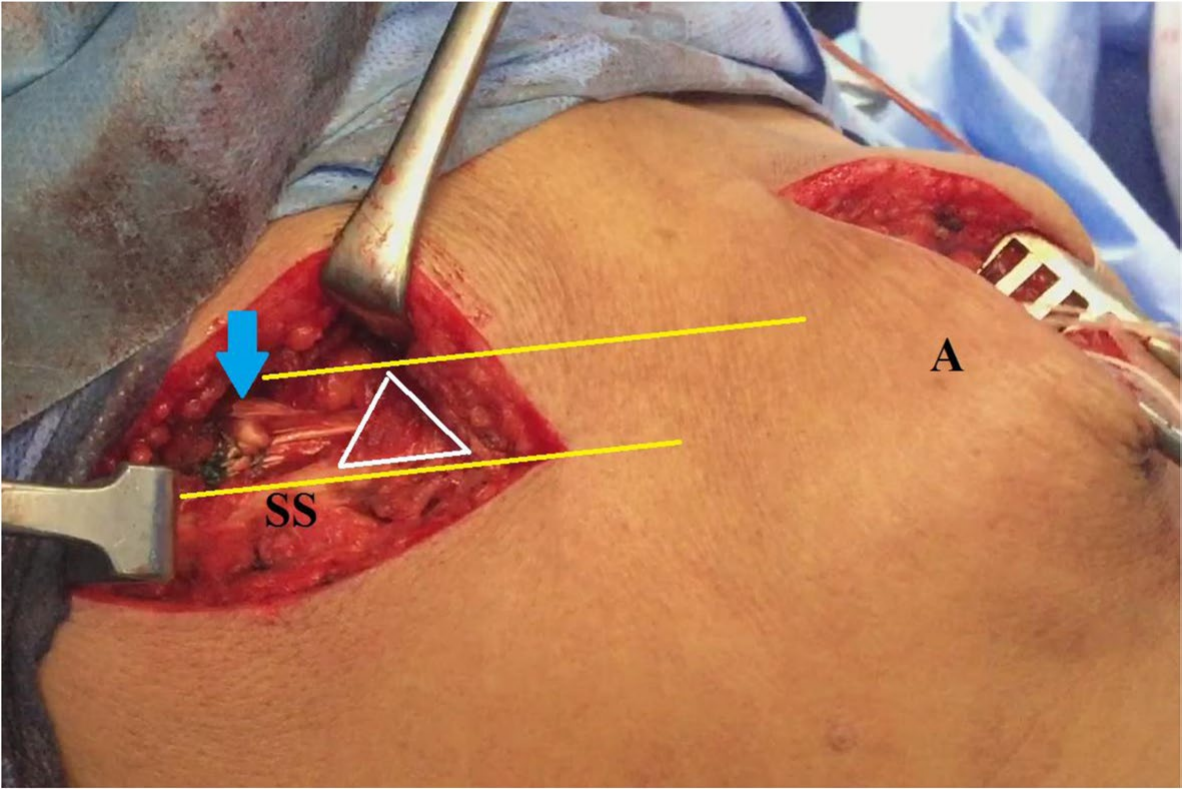
demonstrates passage of the blue arrow-marked tendon reconstruct deep to intact lateral part of the middle trapezius insertion (defined in while triangle) prior to its passage under the acromion; and also demonstrates anatomic convenience of supraspinatus fossa (defined between yellow lines) for reproduction of supraspinatus muscle function by the transferred middle trapezius tendon in the right shoulder; A, acromion; SS, scapular spine

From a biomechanical perspective, MTTT has the potential to almost re-normalize GH kinematics (which in turn, is to pave the way for sustained long-term improvement in GH functional outcomes) via the following mechanisms:

1. Independently of SSP muscle status, MTTT offers nearly-anatomic dynamic reconstitution of SSP function via its close anatomic features and almost collinear force vector to those of SSP muscle, and its phasic recruitment during shoulder elevation [13, 19].
2. When dynamically tested (either in protracted or retracted scapular position), the current technique shows no sub-acromial mechanical block of MTTT as the hamstring sheet does not pass directly deep to the acromion; but instead, it firstly travels deep to intact lateral portion of the middle trapezius insertion prior to its sub-acromial passage. Furthermore, pre-existing extensive SSP atrophy enables SSP fossa to offer a capacious anatomically-convenient room for free gliding motion of MTTT. Figure 13 demonstrates sub-trapezius/sub-acromial passage of the tendon reconstruct and anatomic convenience of SSP fossa for reproduction of SSP function by middle trapezius tendon transfer [13].

Moreover, in current description of MTTT; intra-operative establishment of widened sub-trapezius/sub-acromial track and dynamic testing are essential pearls to ensure smooth and free gliding motion of the tendon reconstruct.

**Fig. 14:**
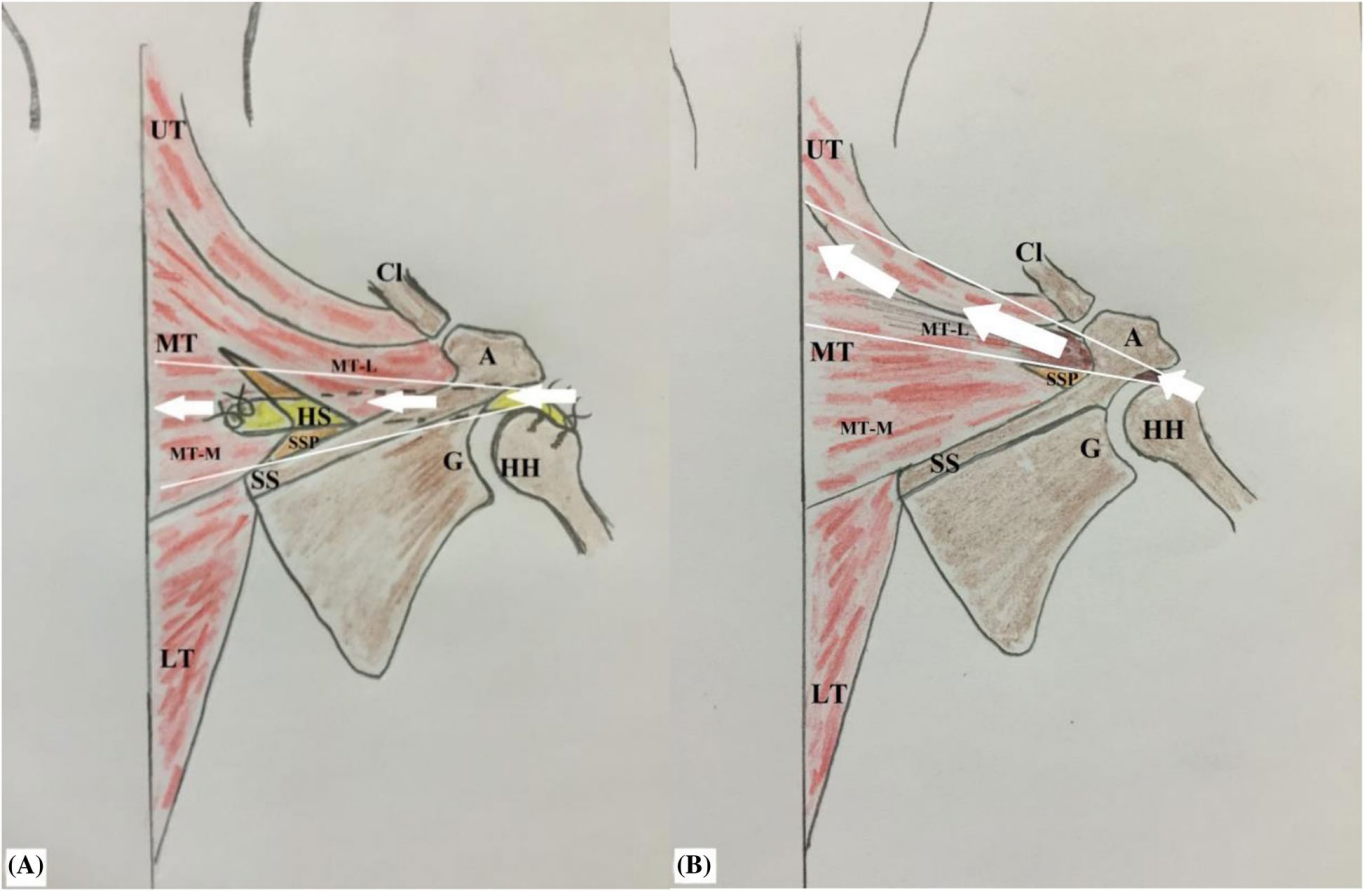
**A, −B** demonstrate direction of force vector of transferred medial portion of the middle trapezius of the current technique in the right shoulder. Compared with that of Moroder et al. of transfer of acromial/lateral portion of the middle trapezius insertion, force vector in currently reported technique is more-collinear with that of supraspinatus muscle; A, acromion; C, coracoid; Cl, clavicle; G, glenoid, HH, humeral head; HS, hamstring sheet; LT, lower trapezius; MT, middle trapezius; MT-L, middle trapezius (lateral insertion); MT-M, middle trapezius (medial insertion); SS, scapular spine; SSP, supraspinatus muscle (atrophied)

Kapicioglu et al., in a letter of the editor, interpreted sub-acromial block of the humeral head reported by Moroder et al.; by superior migration of the humeral head due to vertical/superior direction of force vector of transferred acromial portion of the middle trapezius. This is not the case in the current technique as the transferred portion has a force vector of relatively more horizontal/medial direction; therefore, it might offer a solution to overcome this block. Figure 14A, −B demonstrate direction of force vector of transferred medial portion of the middle trapezius of the current technique compared to that of Moroder et al. [11, 13]

3- Effective action of MTTT necessitates stabilized GH fulcrum; the latter can be achieved via sound force couple mechanism (in the transverse plane) of intact/repaired ISP and SSC (i.e. needed to compress/centralize the humeral head over the glenoid and to counteract deltoid shear force).
4- Current tendon transfer might act as a humeral head depressor via its sub-acromial spacer effect; and additionally, via its tension (i.e. countervailing force of sub-acromially routed hamstring sheet).

Furthermore, SSP is better to be concurrently tension-free (anatomically/partially/medially) repaired in order to gain advantage of its static role in restraining superior migration of the humeral head. This remark might be supported by Burkhart et al. who reported satisfactory functional outcomes of partial and/or medialized RC repair [1].

5- Transferring a small portion of its middle segment while leaving its upper and lower segments un-violated is to keep force couple mechanism of the trapezius required for sound motion of scapula-thoracic articulation.

On other hand, long-term sustainability of improved functional outcomes is a major concern in tendon transfer techniques not able to restore sound GH biomechanics (notably, centralized head over the glenoid). In a study of 108 patients with irreparable RC tear managed by latissimus dorsi transfer, El-Azab et al. reported that 9.3 years-postoperative significant improvement in functional outcome measurements was overshadowed by significantly-progressive decreasing acromio-humeral distance (i.e. superior migration of the humeral head) and increasing RC arthropathy [6, 9, 15].

*.*Meanwhile from a biological perspective, the middle trapezius segment is characterized by enriched vasculature which in turn is to enhance local biology for healing of tendon-tendon interface [18].

***.***Currently described MTTT offers advantages of technical simplicity and reproducibility because of familiarity of orthopedic surgeons with most of its technical steps (e.g., hamstring tendon harvesting and fashioning, Pulvertaft fashion of repair). In addition, easily-palpable scapular spine facilitates identification and harvesting of the middle trapezius.

Compared with that of Moroder et al., current description is technically safer; as it doesn’t violate acromio-clavicular joint for maximizing length of the harvested trapezius tendon. Besides, interposed hamstring sheet is to effectively lengthen the transferred tendon of the middle trapezius; thus, lessening sub-periosteal dissection of the trapezius, reducing tension over the tendon reconstruct and minimizing displacement of trapezius neuro-vascular bundle [8, 13, 15].

.Likewise, current description of MTTT heralds potential technical modifications as feasibility of arthroscopic (i.e., less invasive) humeral re-attachment of the tendon reconstruct and use of tendon allograft instead of hamstring sheet. These potential modifications are to shorten operative time and to minimize related complications [4, 16, 17].

.It is needless to point out that the current technique of MTTT can be performed in concurrence with other GH reconstructive procedures as latissimus dorsi transfer (for restoration of ER). Also, extra-articular soft arthroscopic Latarjet technique (ex-SALT) can be conjugated with MTTT for management of irreparable of RC tear on top of acute dislocation of non-arthritic GH joint in the elderly [10, 17].

.Electro-physiologically, concurrent phasic activity (i.e., synergetic recruitment) of the middle trapezius and SSP during GH elevation might allow accelerated postoperative rehabilitation; which is crucial for negation of formation of sub-trapezius/sub-acromial adhesion [2, 19].

.Current technical note is not without limitations. First, sub-trapezius dissection and roomy SSP fossa might incite seroma formation. Additionally, risk of postoperative infection might be relatively higher due to longer operative time, multiple surgical incisions, abundance of sebaceous cysts (i.e., habitats for P. acne) in the skin overlying the scapula, larger bulk of reconstructed/repaired tissues, more suture volume and implanted hardware, and possibility of undiagnosed (latent) low-grade infection in revision cases.

In addition, lengthy tendon reconstruct and multiple (tendon-tendon/tendon-bone) interfaces might over-burden local biology for healing; predisposing MTTT for failure. The latter risk should be minimized by appropriate techniques of tendons harvesting, preparation and suturing, and meticulous preparation of RC footprint.

Moreover, as it is a lately-evolving technique; MTTTT requires conduction of further biomechanical (e.g. force magnitude and vector), electro-physiological (e.g. activation pattern) and long-term cohort clinical studies to crystallize its validation in management of RC irreparability/re-tear. Besides, consequences of MTTT on nearby cervical spine mechanics should be clarified notably in the light of altered kinetics across the related motion segments. Advantages and disadvantages of the currently reported technique are summarized in Table 4**.**

**Table 4:** Summarizes advantages and disadvantages of currently reported technique of medial trapezius tendon transfer; *AC* acromio-clavicular joint, *ex-SALT* extra-articular soft arthroscopic Latarjet technique, *GH* gleno-humeral, *RC* rotator cuff, *SSP* supraspinatus

**Advantages:**	
∙ More-anatomic dynamic reproduction of SSP function ∙ Re-normalized GH kinematics (centralized humeral head over the glenoid) ∙ Avoidance of mechanical block of the tendon reconstruct ∙ Effective reproduction of anatomic RC footprint ∙ Preserved scapular kinematics ∙ Versatile indications ∙ Technical simplicity, familiarity, safety & reproducibility ∙ Interposed hamstring sheet is to minimize sub-periosteal trapezius dissection, tension over the reconstruct and displacement of related neuro-vasculatures ∙ No violation of AC joint ∙ Feasibility of arthroscopic-assisted modality of the technique ∙ Feasible concurrent GH reconstructive procedures (e.g. latissimus dorsi transfer, ex-SALT) ∙ Accelerated postoperative rehabilitation ∙ Relatively-easy revision	
**Limitations:**	
∙ Possible seroma formation ∙ Relatively higher risk of post-operative infection ∙ Technical irreproducibility in trapezius muscle paralysis ∙ Multiple re-attachment interfaces ∙ No biomechanical validation ∙ No related electrophysiological evidence ∙ No long-term cohort clinical studies ∙ Unclear biomechanical consequences on nearby cervical spine	

## Conclusion

Due to its potential biomechanical, biological and technical advantages, the current technical description of hamstring tendon augmented-middle trapezius tendon transfer (to supraspinatus footprint) can effectively reproduce supraspinatus function; thus, offering a more convenient tendon transfer management option for rotator cuff irreparability/re-tear in young active population of high functional demands.
